# Effect of *Pomacea canaliculata* on *Limnodrilus hoffmeisteri*: Behavior, Oxidative Stress, and Microbiota Alterations

**DOI:** 10.1002/ece3.70603

**Published:** 2024-11-25

**Authors:** Mingyuan Liu, Changrun Sui, Baolong Wang, Pengfei Ma, Weixiao Zhang, Ruipin Huang, Yuqing Wang, Zhujun Qiu, Wenyu Zhao, Tao Zhang, Qian Zhang, Ying Liu

**Affiliations:** ^1^ School of Life Science Liaoning Normal University Dalian China; ^2^ Key Laboratory of Environment Controlled Aquaculture (Dalian Ocean University) Ministry of Education Dalian China; ^3^ College of Fisheries and Life Science Dalian Ocean University Dalian China; ^4^ College of Marine Science and Technology Dalian Ocean University Dalian China; ^5^ College of Biosystems Engineering and Food Science Zhejiang University Hangzhou China

**Keywords:** *Aeromonas*, benthic worm, intestinal microbiota, invasion mechanism, *Pomacea canaliculata*

## Abstract

*Pomacea canaliculata* is an invasive species which has significantly impacted native ecosystems globally. The benthic worm *Limnodrilus hoffmeisteri* is essential for the stability of the native aquatic ecosystem, facilitating the nutrient cycle dynamics through bioturbation. Nevertheless, limited information exists regarding the impact of *P. canaliculata* on those key native benthic species. Present study evaluated the impacts of *P. canaliculata* on *L. hoffmeisteri* by exposing *L. hoffmeisteri* to *P. canaliculata* (PC group) and the native snail *Bellamya aeruginosa* (BA group), with a control group consisting of no snails (NS group). The survival rate of *L. hoffmeisteri* in the PC group persisted diminished over 14 days, with notable declines in the rates of successful food acquisition and aggregation, an increase in migration, and a decrease in swing frequency. Elevated oxidative stress levels were linked to these alterations in *L. hoffmeisteri* behavior. Additionally, the presence of *P. canaliculata* increased the abundance of intestinal pathogenic bacteria in *L. hoffmeisteri* , with *Aeromonas* being one of the most lethal. Experimental models of *Aeromonas*‐free *P. canaliculata* (AFPC), re‐infected AFPC (IPC), and *Aeromonas* (As) were established to illustrate the role of *Aeromonas* in the decline of *L. hoffmeisteri* . Similar patterns in *L. hoffmeisteri* survival, behavior, and oxidative stress were observed in As, IPC, and PC group; however, these effects were mitigated by the elimination of *Aeromonas* in the AFPC group. Furthermore, *L. hoffmeisteri* was fatally affected by the four *Aeromonas* strains that were obtained from *P. canaliculata* intestine. These findings indicate that *P. canaliculata* exerts a deleterious impact on *L. hoffmeisteri* , and *Aeromonas* colonizing in intestine plays an important role. This study reveals a novel invasion mechanism of *P. canaliculata* .

## Graphical abstract

Behavior pattern changes of *L. hoffmeisteri* exposed to non‐free *Pomacea canaliculata* (PC) were found PC caused oxidative stress in *L. hoffmeisteri* PC changed the intestinal microbiota community structure of *L. hoffmeisteri* *Aeromonas* from the intestinal tract of *P. canaliculata* (As) had similar effects. As is the main stress source.

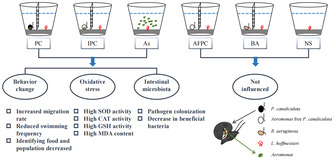

## 1 Introduction

Biological invasions have become a prominent environmental concern in the 21st century, leading to profound economic losses and ecosystem disruptions (Simberloff et al. 2013). In China, invasive species have been widely documented across diverse ecosystems, exerting widespread, and persistent impacts (Oya, Hirai, and Miyahara 1987; Susin 2004). The process of biological invasion is a dynamic and complicated phenomenon, the success of invasion depends not only on the biological characteristics of the invasive species, but also on the ecological environment of the habitat (Alpert, Bone, and Holzapfel 2000). Mounting evidence points that complex non‐additive interaction effects exist among the drivers contributing to the decline of native species. Clearly, invasive species are one of the direct causes of biodiversity loss; however, the relationship between invasive and native species is not simply one of trade‐offs. In fact, invasive species may lead to environmental changes in habitats, resulting in significant indirect impacts on native species (Didham et al. 2005, 2007), they can affect native species directly by predation or competition resource within the ecosystem, or indirectly affect native species by secreting allelochemicals or microorganisms. For instance, native tadpoles have to adapt to the substances secreted by the invasive crayfish ( *Procambarus clarkii* ), potentially leading to phenotypic changes in the population over time due to heightened stress (Melotto, Manenti, and Ficetola 2020). Furthermore, the biological composition such as microbial communities and phytoplankton communities changes in the native ecosystem were observed in response to the presence of the invasive phytophagous fish *Siganus rivulatus* , as evidenced by alterations in the nutrient content of its feces and other released materials (Escalas et al. 2022). It has been reported that native species may also be influenced by visual cues from invasive predators, causing them to remain in a prolonged state of alertness, which reduces their feeding activities and results in population decline (Nunes et al. 2013).

*Pomacea canaliculata* Lamarck, 1819 (Neogastropoda, Ampullariidae), commonly known as the golden apple snail, is a highly invasive aquatic species that has established a strong presence not only in China but also in numerous other regions around the world. It is remarkable adaptability and prolific reproductive capacity enabled it to invade a wide range of freshwater ecosystems in China (Oya, Hirai, and Miyahara 1987; Susin 2004). *P. canaliculata* is an omnivorous snail with a preference for plants consumption, which poses a substantial menace to rice crops. Additionally, it preys on native snails and other aquatic animals such as *Paramecium caudatum* , disrupting the ecological balance and, consequently, endangering the biodiversity of aquatic ecosystems (Carlsson, Brönmark, and Hansson 2004; Escalas et al. 2022; King‐Lun, Chan, and Qiu 2009; Matsukura et al. 2016). Moreover, *P. canaliculata* indirectly influences the density of primary producers and the primary productivity of the entire ecosystem by consuming algae and phytoplankton (Ling et al. 2010). Furthermore, the metabolites and excreta of *P. canaliculata* contribute to increase in nitrogen and phosphorus content, stimulating the proliferation of microorganisms and leading to the water quality deterioration or eutrophication (Carlsson, Brönmark, and Hansson 2004; O'Neil et al. 2023). It has also been reported that the secretions of *P. canaliculata* directly impact the structure of bacterial communities and the survival of adjacent species (Maldonado and Martín 2019; Wang et al. 2020).

Bioturbation is a crucial process in the modification of material cycling and energy flow within the sediment/water interface and is conducted benthic organisms through various activities such as feeding, excavation, and exploration (Lohrer, Thrush, and Gibbs 2004). These activities profoundly influence the physical and chemical properties of both the sediment and water (Pratihary et al. 2009). The bioturbation rate varies among different functional groups of macroinvertebrates, with Tubificidae exhibiting a notably stronger bioturbation ability compared to other benthic animals (Michaud et al. 2005, 2006). *Limnodrilus hoffmeisteri* Claparède, 1862 (Tubificida, Tubificidae) is the most abundant freshwater worms, it dominates in many freshwater environments throughout China (Zhang et al. 2010). The swing movement of *L. hoffmeisteri* facilitates oxygenation, nesting and feeding, resulting in the transportation and mixing of sediment particles, as well as the changing the pH, oxidation–reduction potential and dissolved oxygen content of sediment–water interface (Zhang et al. 2010). Therefore, *L. hoffmeisteri* plays a pivotal role in maintaining the stability of the aquatic environment. Nevertheless, the widespread distribution of *L. hoffmeisteri* has led to its dominance in water body invaded by the *P. canaliculata* . However, the effect of the invasion of *P. canaliculata* on the population of *L. hoffmeisteri* remains unclear.

Throughout the extensive history of co‐evolution between hosts and microorganisms, the intestinal microbiota has progressively established a mutually cooperative and symbiotic relationship with the host organism. In usual conditions, the intestinal microbiota maintains a dynamic balance state in response to complex and changeable environments. This balance is crucial for the host organism's assimilation and metabolism of nutrients, as well as the regulation of its immune response (Arthur et al. 2013; Fassarella et al. 2021; Yang, Yang, and Li 2022). However, the intestinal microbiota of aquatic invertebrates is highly susceptible to environmental changes, providing a potential gateway for pathogen such as *Aeromonas* invasion and colonization within the intestine (Chen et al. 2021; Chen et al. 2008; Wang et al. 2019). The primitive immune system of *L. hoffmeisteri* heavily relies on the assistance of its intestinal microbiota, making it vulnerable to pathogenic bacteria, including *Aeromonas*, which colonize the intestine of *P. canaliculata* (Chen et al. 2021; Li et al. 2022). The release of these pathogenic bacteria to the aquatic environment may potentially result in infections, illness, and even death among other aquatic organisms.

This study focused on the non‐predatory interactions between *P. canaliculata* and the key native bioturbator‐ *L. hoffmeisteri* . To investigate the effects and underlying mechanisms of *P. canaliculata* invasion on native bioturbator, the behavioral reactions, oxidative stress levels, and alterations in the intestinal microbiota of *L. hoffmeisteri* in response to *P. canaliculata* and native snail *Bellaya aeruginosa* exposure were analyzed. *Aeromonas* represents one of the most abundant bacterial communities in the gut of *P. canaliculata* , may enter aquatic ecosystems through the secretion and excretion processes of *P. canaliculata* , potentially leading to infections and mortality among *L. hoffmeisteri* . We constructed an *Aeromonas*‐free *P. canaliculata* model (AFPC) and an *Aeromonas*‐infected AFPC model (IPC) to further clarify the significance of *Aeromonas* from the gut of *P. canaliculata* by exposing *L. hoffmeisteri* to the two animal models. At last, we isolated three *Aeromonas* strains from the gut of *P. canaliculata* and exposed *L. hoffmeisteri* to these strains to demonstrate the direct pathogenicity of the *Aeromonas* isolates present in the invasive snail's gut. Our findings establish a theoretical basis for evaluating the ecological dangers posed by the invasion of *P. canaliculata* on bioturbators and clarify the probable pathways by which the gut microbiota of *P. canaliculata* affects these interactions. This method enriches the theoretical evidence that an interaction modification effect between invasive species and native species.

## 2 Materials and Methods

### 2.1 Test Organism

The invasive snail *Pomacea canaliculata* was collected from a lake in Zhaoqing (Guangdong Province, China). The native snail *Bellaya aeruginosa* Reeve, 1863 (Mesogastropoda, Viviparidae), which is the near niche species of *P. canaliculata* in China, is a wild population in Weishan Lake obtained from local fishermen, which are set as control group (Shandong Province, China). *Limnodrilus hoffmeisteri* were collected from the sediment of Dalian Qianguan Wetland Park (Liaoning province, China). No natural contact occurred between the populations of these three animals. All animals were immediately transported to the laboratory under cool conditions.

The snails were kept in a 76 L water tank containing 50 L aerated tap water, accommodating 100 animals per tank. And *L. hoffmeisteri* were kept in 3.5 L water tanks with 3 L aerated tap water, containing approximately 1000 ind per tank. All species were acclimated in deionized water separately, and maintained under a 12:12 light/dark (L:D) cycle at a temperature of 20°C ± 2°C for a period over 14 days. All animals were fed with homogeneous commercial fish food (Tetramin, Germany) at a rate of 0.1 mg/ind/d.

### 2.2 Establishment of Animal Model

Our previous study has demonstrated that *Aeromonas* from the intestine of *P. canaliculata* plays a crucial role in the infection of native snails (Data not published). Four major *Aeromonas* strains were isolated from the intestinal tract of *P. canaliculata* : *Aeromonas* sp., *Aeromonas hydrophila* , *Aeromonas media* , and *Aeromonas veronii* . These strains were sequenced and preserved in the laboratory. For the present study, a mixed *Aeromonas* culture was created by blending the four strains in equal proportions (1:1:1:1) in the *L. hoffmeisteri* infection group (As).

To establish the *Aeromonas*‐free *P. canaliculata* model (AFPC), *P. canaliculata* individuals were cultured at a density of 4 ind/L and treated with florfenicol at a concentration of 2 g/L for 4 days, with the treatment solution renewed daily. The successful construction of the AFPC model was confirmed by the absence of *Aeromonas* colonies in the microbial culture of the AFPC intestinal tract. All the snails survived after treatment, and the behavior patterns did not change.

To establish the *Aeromonas*‐infected AFPC model (IPC), the established AFPC were exposed to a culture of mixed *Aeromonas* at a concentration of 1 × 10^8^ CFU/mL for 24 h. The establishment of the IPC model was confirmed by the detection of *Aeromonas* colonies in the microbial culture of the IPC intestinal tract.

### 2.3 Experimental Design

For the non‐contact exposure experiment, round polyethylene box (1250 mL) with small polyethylene boxes with holes inside, allowing water circulation and keeping the animals isolated was used as the experimental system. Sixty experimental systems were established and randomly divided into three groups: the invasion group with one *P. canaliculata* (PC) (7.00 ± 0.30 g), the native group with one *B. aeruginosa* (BA) (2.50 ± 0.20 g), and the control group without snails (NS). Initially, the bottom of the round polyethylene box was layered with sterilized and cooled fine river sand (diameter 1–2 mm) at a thickness of 1.5 cm. Subsequently, 800 mL of aerated tap water and 1000 ind *L. hoffmeisteri* individuals were introduced into the system after acclimation for 24 h. To further assess the impact of gut microbiota from *P. canaliculata* on *L. hoffmeisteri*. One of the representative model animals from AFPC group, IPC group and As group were introduced into the small polyethylene boxes. And the other experimental settings kept the same.

The two experiments lasted for 14 days. At D7 and D14, 10 repetitions systems from each experimental group were collected for subsequent analysis. *L. hoffmeisteri* were isolated from the sand and their survival rates were counted, then the behavioral indicators and antioxidant enzymes activity were assessed. *L. hoffmeisteri* individuals form the PC, BP, and NS group were collected for 16S rRNA microbiota sequencing.

### 2.4 Survival Rate of *L. hoffmeisteri*

At D7 and D14, 10 experimental systems of PC, BP, NS and AFPC, IPC, AS group were sampled, following which the bottom sediments were thoroughly transferred onto a white porcelain plate, all the *L. hoffmeisteri* were isolated from the sand. Subsequently, all surviving *L. hoffmeisteri* were identified and quantified. The survival rates for each group at D7 and D14 were calculated.

### 2.5 Behavior Responses of *L. hoffmeisteri*

*L. hoffmeisteri* typically form a large nucleus population, and individual worms exhibit migration behaviors to avoid potentially hazardous environmental conditions. Thus, the migration patterns of *L. hoffmeisteri* from the nucleus population can serve as an indicator for assessing environmental conditions, and their swing behavior can be used to evaluate their vitality.

A total of 500 *L. hoffmeisteri* individuals were collected from all experimental groups at D7 and D14. These worms were placed in a 6‐well cell culture plate filled with 10 mL of deionized water per well. Worms observed swinging alone far away from the nucleus population, or those formed a small population, were categorized as migratory individuals. After holding for 24 h, the proportion of migratory *L. hoffmeisteri* in total *L. hoffmeisteri* was observed in 10 wells. The swing frequency of *L. hoffmeisteri* from migratory individuals or nucleus population individuals was then recorded within 1 min. The behavior of 20 randomly selected *L. hoffmeisteri* was observed from at least five wells, and the average number was recorded.

The acquisition of food is an innate reflexive behavior of *L. hoffmeisteri* , with the speed of exploring and capturing food reflecting their vitality and physiological state. To investigate the effects of the treated groups on this behavior, 500 *L. hoffmeisteri* individuals were collected and placed in the center of a well. A piece of fish food was placed on the opposite edge of the well. The time it took for the individuals to extend out of their bodies and retrieve the food to the central location was recorded as a successful food capture event. Food acquisition rates were calculated at different time points, and the NS, BA and PC group was repeated for six times at D7 and 10 times at D10. The AFPC, As, and IPC group was repeated for 10 times at both D7 and D10.

*L. hoffmeisteri* individuals tend to aggregate into large nucleus populations. However, their ability to recognize population boundaries might be influenced by environment deterioration or exposure to stressors. To assess this, *L. hoffmeisteri* collected from the different experimental groups on D7 and D14 were placed on the leftmost and rightmost edges of a culture plate, with 250 individuals on each side. The time taken for the two populations to aggregate into one nucleus population was recorded, and the NS, BA, and PC groups were repeated six times at D7 and 10 times at D10. The AFPC, As, and IPC groups were repeated 10 times on both D7 and D10.

### 2.6 Antioxidant Enzyme Activity Detection

To assess the oxidative stress in *L. hoffmeisteri* at D7 and D14, 200 *L. hoffmeisteri* individuals from each experimental group were collected in experimental systems triplicate. The collected samples were homogenized with physiological saline, followed by centrifugation at 2500 rpm/min at 4°C for 10 min. The supernatant was collected to detect malondialdehyde (MDA), superoxide dismutase (SOD), catalase (CAT), and reduced glutathione (GSH) following the instruction of the commercial assay kits (Nanjing Jiancheng Bioengineering Institute, Nanjing, China). Additionally, protein concentrations were determined using the bicinchoninic acid (BCA) protein kit as standard.

### 2.7 Intestinal Microbiota Analysis

*L. hoffmeisteri* were fasted for 24 h and cleaned before sampling. The intestinal contents were extracted, immediately frozen in liquid nitrogen, and stored at −80°C until use. All samplings in the experiment were performed under sterile conditions. For the NS, BA, and PC groups at D7 and D14, *L. hoffmeisteri* were washed three times with sterile physiological saline, and 150 worms were pooled into a single sample. The total genome DNA were extracted and the DNA concentration and purity were measured. Then the bacterial 16S rRNA gene V4 region was amplified with the primers 515F‐806R (F: CCTAYGGGRBGCASCAG, R: GGACTACNNGGGTATCTAAT). The amplified DNA was subsequently sequenced on an Illumina MiSeq platform of Novogene (Beijing, China), generating 250 bp paired‐end reads for intestinal microbiota analysis. The raw sequencing data is assembled and filtered to obtain Clean Data; then denoising is performed using DADA2 based on the Clean Data, filtering out sequences with abundances lower than five to obtain the final ASVs.

### 2.8 Acute Toxicological Experiment of *Aeromonas* on *L. hoffmeisteri*

The cumulative mortality rate of *L. hoffmeisteri* with the exposure to *Aeromonas* from the intestinal tract of *P. canaliculata* was investigated. *Aeromonas* sp., *Aeromonas hydrophila* , *Aeromonas media* , and *Aeromonas veronii* , and the mixed *Aeromonas* in a 1:1:1:1 ratio of four *Aeromonas* with a concentration of 10^8^ CFU/mL were used as experimental bacteria. A six‐well cell culture plate was used as the experimental system. Ten individual *L. hoffmeisteri* without any prior experimental treatment were placed into the wells. The control group (CK) received sterile water only. Repeat six times in each group. All the surviving *L. hoffmeisteri* individuals were observed, and the cumulative mortality rate of *L. hoffmeisteri* was calculated.

### 2.9 Statistical Analysis

Data were presented as mean ± standard deviation (SD) and tested for statistical significance using Paired‐sample *t*‐tests. The statistical values were considered significantly different when the calculated probability (*p*) level was below 0.05. The results were plotted using GraphPad Prism 9.

## 3 Results

### 3.1 Effects of the *P. canaliculata* on the Survival Rate and the Behavior of *L. hoffmeisteri*

**FIGURE 1:**
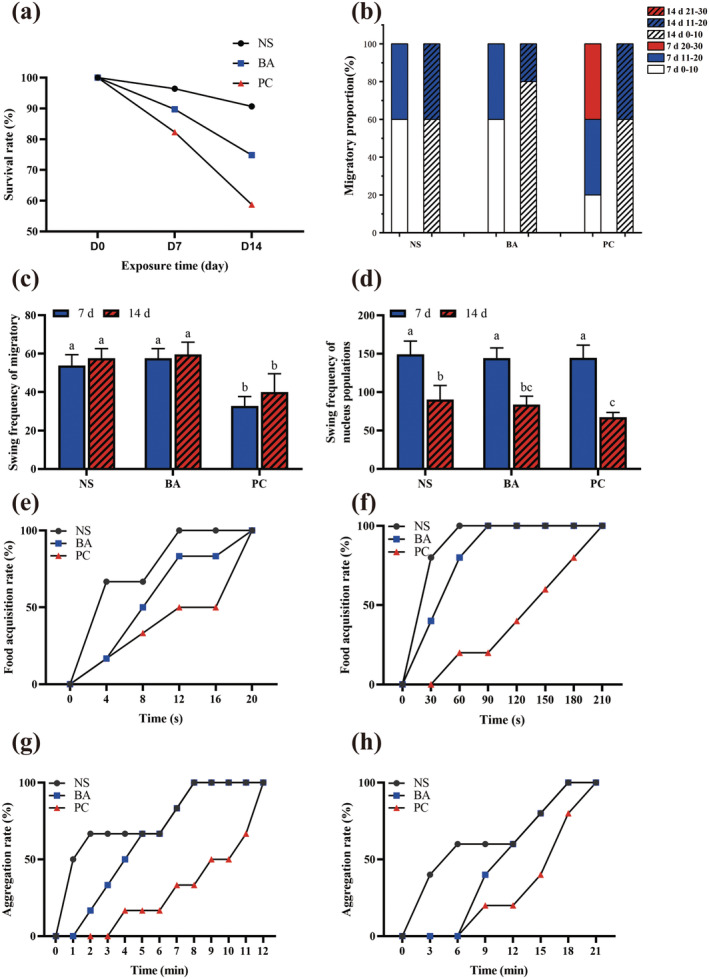
The survival rate and behavior of *Limnodrilus hoffmeisteri* after different treatments. (a) The survival rate of *L. hoffmeisteri* ; (b) Proportion of the migratory numbers of *L. hoffmeisteri* ; (c) Swing frequency of migratory *L. hoffmeisteri* ; (d) Swing frequency of nucleus populations *L. hoffmeisteri* ; (e) Successful food acquisition rate of *L. hoffmeisteri* at D7; (f) Successful food acquisition rate of *L. hoffmeisteri* at D14; (g) Aggregation rate of *L. hoffmeisteri* at D7; (h) Aggregation rate of *L. hoffmeisteri* at D14. BA: *B. aeruginosa*; NS: None snails; PC: *P. canaliculata* . Different superscript letters indicate significant difference between groups (*p* < 0.05).

The survival rate of *L. hoffmeisteri* in all experimental groups exceeded 80% at D7 (Figure 1a). Specifically, the NS group, BA group, and PC group exhibited survival rates of 96.4%, 89.7%, and 82.28%, respectively. With the extension of treatment duration, the survival rates of *L. hoffmeisteri* decreased to 90.68%, 74.8%, and 58.71% at D14, respectively. It is worth noting that the PC group exhibited significantly lower survival rate compared to the BA group and NS group at both D7 and D14 (*p* < 0.05).

It was observed that exposure to *P. canaliculata* had an irritating effect on the behavior pattern of *L. hoffmeisteri* . Most of the wells in NS and BA groups had only 0–10 migratory individuals at D7 (Figure 1b). Conversely, the PC group displayed more pronounced migration, with 40% of the experimental units exhibiting 21–30 migratory individuals, and 40% having 11–20 migratory individuals. The findings suggest that *P. canaliculata* elicits alarm response in *L. hoffmeisteri* . Furthermore, the proportion of highly migratory individuals of *L. hoffmeisteri* in the PC group exhibited a significant decrease at D14. Only 20% of the wells displayed 11–20 migratory individuals, indicating that prolonged exposure to *P. canaliculata* has fostered adaptability in *L. hoffmeisteri* , resulting in reduced vigilance behavior.

The migratory *L. hoffmeisteri* individuals in the PC group exhibited a significantly lower swing frequency compared to those in the NS and BA group at both D7 and D14 (*p* < 0.05) (Figure 1c). Similarly, the swing frequency of nuclear *L. hoffmeisteri* in the PC group was significantly lower than that in the NS group at D14 (*p* < 0.05) (Figure 1d), while no significant difference was observed in the swing frequency of nuclear *L. hoffmeisteri* among all groups within the nucleus population at D7 (*p* > 0.05). Therefore, the invasion of *P. canaliculata* will persistently exert a negative impact on the bioturbation performance of *L. hoffmeisteri* .

In terms of food acquisition, the rate of food acquisition of *L. hoffmeisteri* in the PC group was the slowest (Figure 1e). Only 50% of the experimental wells in the PC group successfully obtained food within 12 s at D7. In contrast, all the experimental units in the NS group successful obtained food within 12 s, and 50% of experimental units obtained food within 3 s. In the BA group, it took 9 s for 50% of the experimental units to successfully obtain food. As the treatment duration prolonged, the time required for each group to successfully acquire food also increased. In the PC group, the food acquisition rate reached 100% at 210 s at D14 (Figure 1f). These findings indicate that *P. canaliculata* may affect the food acquisition and exploration ability of *L. hoffmeisteri* .

Furthermore, *L. hoffmeisteri* individuals in the PC group encountered challenges in recognizing and aggregating with each other, only 50% of the experimental units successfully aggregated within 9 min at D7, while it took 11 min for all experimental units to achieve successful aggregation. In contrast, *L. hoffmeisteri* in the BA and NS group identified and aggregated with each other within 8 min (Figure 1g). With the extension of experiment duration, the aggregation rate between populations of *L. hoffmeisteri* under different treatments decreased. In the PC group, all the experimental units successfully aggregated within 18 min (Figure 1h), highlighting a diminished population recognition ability of *L. hoffmeisteri* in response to *P. canaliculata* invasion.

### 3.2 Effects of the *P. canaliculata* on the Antioxidant Responses of *L. hoffmeisteri*

**FIGURE 2:**
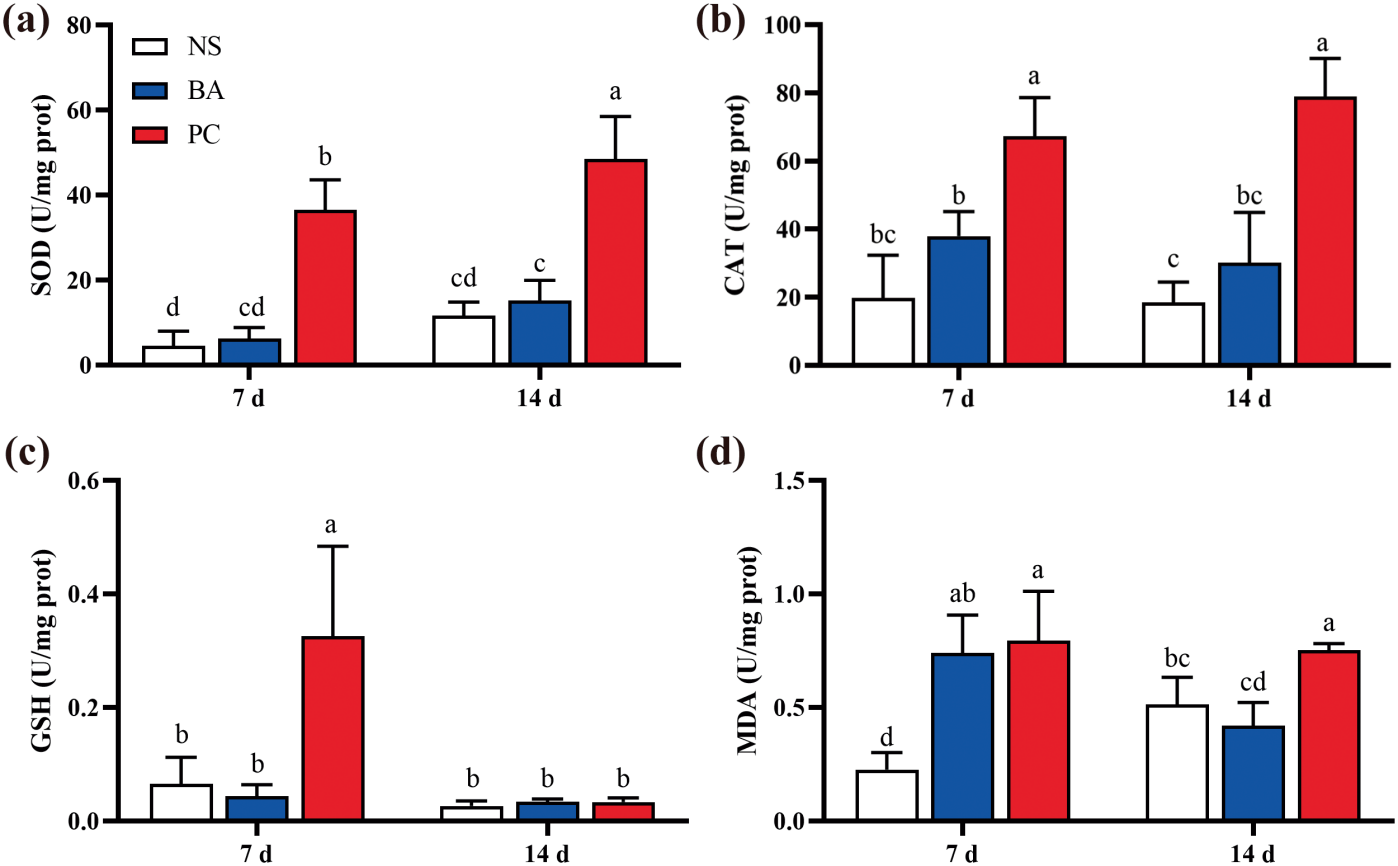
Enzymatic activity and lipid peroxidation of *L. hoffmeisteri* after different treatments. (a) SOD; (b) CAT; (c) GSH; (d) MDA.

The levels of SOD and CAT activity in *L. hoffmeisteri* showed a consistent trend, with significant increases in the PC group compared to the other groups at both D7 and D14 (*p* < 0.05). The SOD activity in the PC group at D14 was significantly higher than that at D7 (*p* < 0.05) (Figure 2a,b). The GSH content in PC group was significantly higher than that both in BA and NS group at D7 (*p* < 0.05), while it inhibited in all groups with no significant differences at D14 (*p* > 0.05) (Figure 2c). Additionally, the MDA content in both PC and BA groups were significantly higher than those in the NS group at D7 (*p* < 0.05), and the MDA content in the BA group was significantly reduced to a level comparable to that of the NS group at D14 (*p* < 0.05), while no significant changes were observed in PC group (Figure 2d).

### 3.3 Effects of the *P. canaliculata* on the Intestinal Microbiota of *L. hoffmeisteri*

For *L. hoffmeisteri* , a total of 2929, 3666, 3735, 1561, 2637, and 2089 ASVs were observed in the NS7d, BA7d, PC7d, NS14d, BA14d, and PC14d groups, respectively. Furthermore, 1168 ASVs (39.88%) were identified in both NS7d and BA7d groups, while 813 ASVs (27.76%) were shared between the NS7d and PC7d groups. Additionally, 809 (51.826%) ASVs were shared between NS14d and BA14d groups, and 403 ASVs (25.82%) were observed in both NS14d and PC14d groups. Moreover, 485 ASVs were identified as common between NS7d and NS14d groups, whereas 1044 ASVs exhibited between BA7d and BA14d groups. Lastly, 547 ASVs were shared by both PC7d and PC14d groups.

**FIGURE 3:**
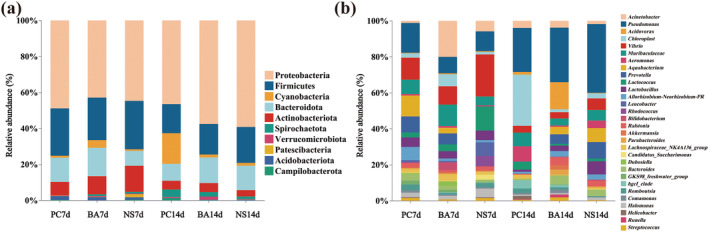
The composition of intestinal symbiotic bacteria of *L. hoffmeisteri* under different treatments. (a) phylum level; (b) genus level.

Both *P. canaliculata* and *B. aeruginosa* had an impact on the intestinal microbial structure of *L. hoffmeisteri* , with the effect of *P. canaliculata* much greater than that of the native snail. The most prevalent bacterial groups in both NS7d and NS14d group were Proteobacteria, Firmicutes, and Bacteroidota at phylum level (Figure 3a). Furthermore, several genera exhibited changes in abundance following different durations of exposure to different snail species. Notably, *Pseudomonas* was found to be one of the dominant bacteria in the intestine of *L. hoffmeisteri* . At D7, the relative abundance of *Pseudomonas* was higher in the group exposed to *P. canaliculata* compared to other groups, while its relative abundance was lower than that of other groups at D14. Exposure to the two snails species resulted in an enrichment of *Aeromonas* in the intestine of *L. hoffmeisteri* , with the relative abundance of *Aeromonas* being particularly high in the groups exposed to *P. canaliculata* . The relative abundance of *Lactobacillus* in different groups remained stable at D7, but its relative abundance significantly decreased in PC14d group. Additionally, the relative abundance of *Rhodococcus* was found to be extremely low in the PC7d group and was not detected in the PC14d group (Figure 3b).

### 3.4 Effects of *Aeromonas* on the Survival Rate and the Behavior of *L. hoffmeisteri*

**FIGURE 4:**
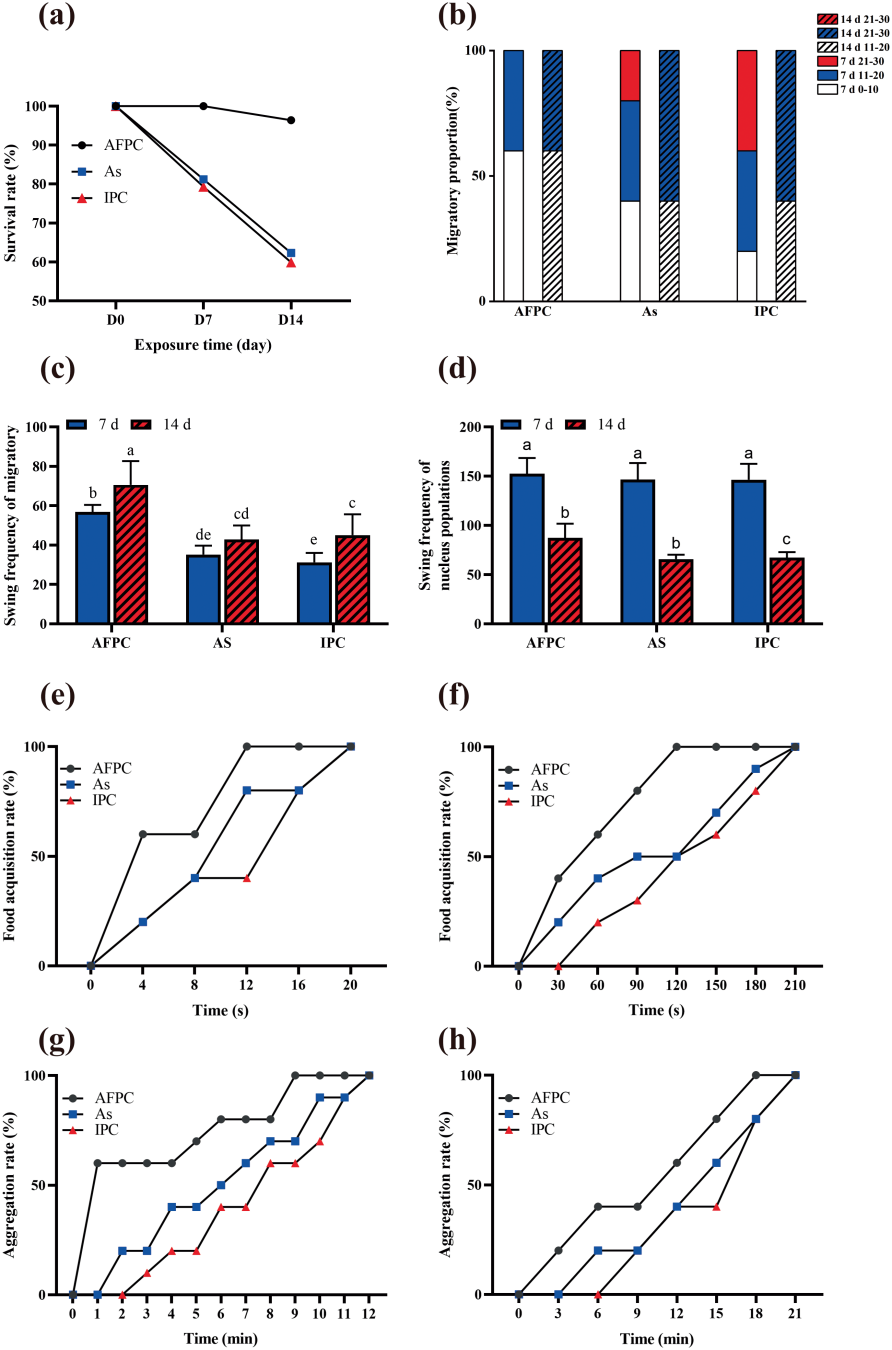
The survival rate and behavior of *L. hoffmeisteri* after different treatments (AFPC, As or IPC). (a) The survival rate of *L. hoffmeisteri* ; (b) Proportion of the migratory numbers of *L. hoffmeisteri* ; (c) Swing frequency of migratory *L. hoffmeisteri* ; (d) Swing frequency of nucleus populations *L. hoffmeisteri* ; (e) Successful food acquisition rate of *L. hoffmeisteri* at D7; (f) Successful food acquisition rate of *L. hoffmeisteri* at D14; (g) Aggregation rate of *L. hoffmeisteri* at D7; (h) Aggregation rate of *L. hoffmeisteri* at D14. Different superscript letters indicate significant difference between groups (*p* < 0.05).

The survival rates of *L. hoffmeisteri* in AFPC group, As group, and IPC group at D7 was 100.0%, 81.2%, and 79.3%, respectively. With the extension of treatment duration, the survival rate of *L. hoffmeisteri* in As group and IPC group at D14 significantly decreased to 62.3% and 59.9%, respectively (Figure 4a).

At D7, the majority of AFPC groups exhibited low migratory activity with 0–10 individuals, while significant migrations were observed in the As and IPC groups (Figure 4b). Specifically, 20% of the experimental units in the As group had 21–30 migratory individuals, and 40% of the experimental units in the IPC group showed a similar trend with high numbers of migratory individuals. This finding suggests that the infection of *Aeromonas* or *P. canaliculata* with *Aeromonas* was more likely to cause alert or hyperactive behavior in *L. hoffmeisteri* . Besides, the proportion of high migratory individuals of *L. hoffmeisteri* in the As and IPC groups decreased at D14. This finding is consistent with previous experimental results in PC group, indicating that with the extension of exposure time, the activity of *L. hoffmeisteri* decreased The swing frequency of migratory *L. hoffmeisteri* individuals in the As and IPC groups was significantly lower compared to the AFPC group at D7 or D14 (*p* < 0.05) (Figure 4c). However, there was no significant difference in the swing frequency of the *L. hoffmeisteri* nucleus population at D7 (*p* > 0.05), but the swing frequency was significantly lower in the IPC group compared to the As and AFPC groups (*p* < 0.05) (Figure 4d). These findings suggest that *P. canaliculata* can cause a decrease in swing frequency and activity of *L. hoffmeisteri* through the secreting of *Aeromonas*.

The presence of *Aeromonas* from *P. canaliculata* 's intestinal, as well as the carriage of *Aeromonas* by *P. canaliculata* , greatly impacts the food exploration and acquisition of *L. hoffmeisteri* . Only 40% of experimental units in the IPC group successfully obtained food within 12 s at D7. In contrast, all experimental units from both the IPC and As groups were able to obtain food within 20 s, while all experimental units in AFPC group 12 s (Figure 4e). With the extension of treatment time, the time required for each group to successfully obtain food increased. In the As and IPC groups, the food acquisition rate reached 100% after a period of 210 s at D14 (Figure 4f).

Furthermore, it was observed that *L. hoffmeisteri* in the As and IPC groups had difficulty in identifying and aggregating with each other. All experimental units in the As and IPC groups successfully aggregated within 12 min, while all units in the AFPC group were identified and aggregated within 9 min (Figure 4g). With the extension of experimental exposure time, the aggregation speed between *L. hoffmeisteri* populations under different treatments decreased. All the experimental units in the As and IPC groups successfully aggregated at 21 min at D14 (Figure 4h), which is consistent with previous experimental results. These results suggest that both the presence of *Aeromonas* from the intestinal of *P. canaliculata* and the presence of *P. canaliculata* carrying *Aeromonas* significantly impact the population identification and aggregation of *L. hoffmeisteri* .

### 3.5 Effects of the *Aeromonas* on the Oxidation, and Antioxidant Responses of *L. hoffmeisteri*

**FIGURE 5:**
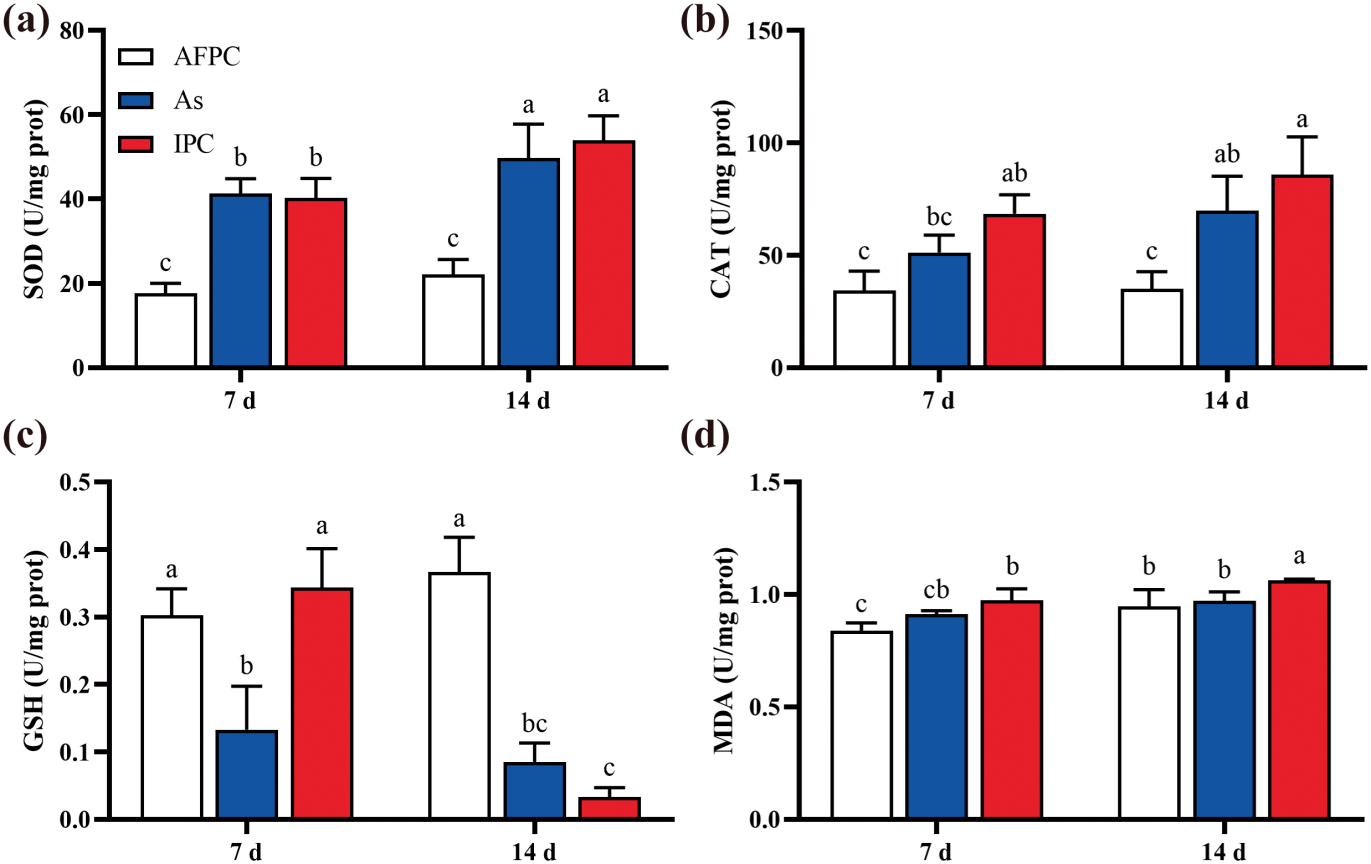
The oxidation and antioxidant responses of *L. hoffmeisteri* after different treatments. (a) SOD; (b) CAT; (c) GSH; (d) MDA.

The SOD activity of *L. hoffmeisteri* in As and IPC groups were significantly higher than that in the AFPC group (*p* < 0.05) both at D7 and D14, with further increased levels observed at D14 (*p* < 0.05) (Figure 5a). The CAT activity of *L. hoffmeisteri* in the IPC group was higher than the As group, and significantly elevated when compared to that in AFPC group at D7 and D14 (Figure 5b). The GSH content in the AFPC group was significantly higher compared to the As and IPC groups at D14 (*p* < 0.05). Furthermore, the content of GSH in IPC group decreased significantly as the experimental duration extended (*p* < 0.05) (Figure 5c). The MDA content in IPC group was significantly higher compared to the AFPC group (*p* < 0.05) at D7 (Figure 5d). Moreover, the MDA content in the IPC group was significantly higher when compared to both the As and AFPC groups at D14 (*p* < 0.05).

### 3.6 Effects of *Aeromonas* on the Cumulative Mortality Rate of *L. hoffmeisteri*

**FIGURE 6:**
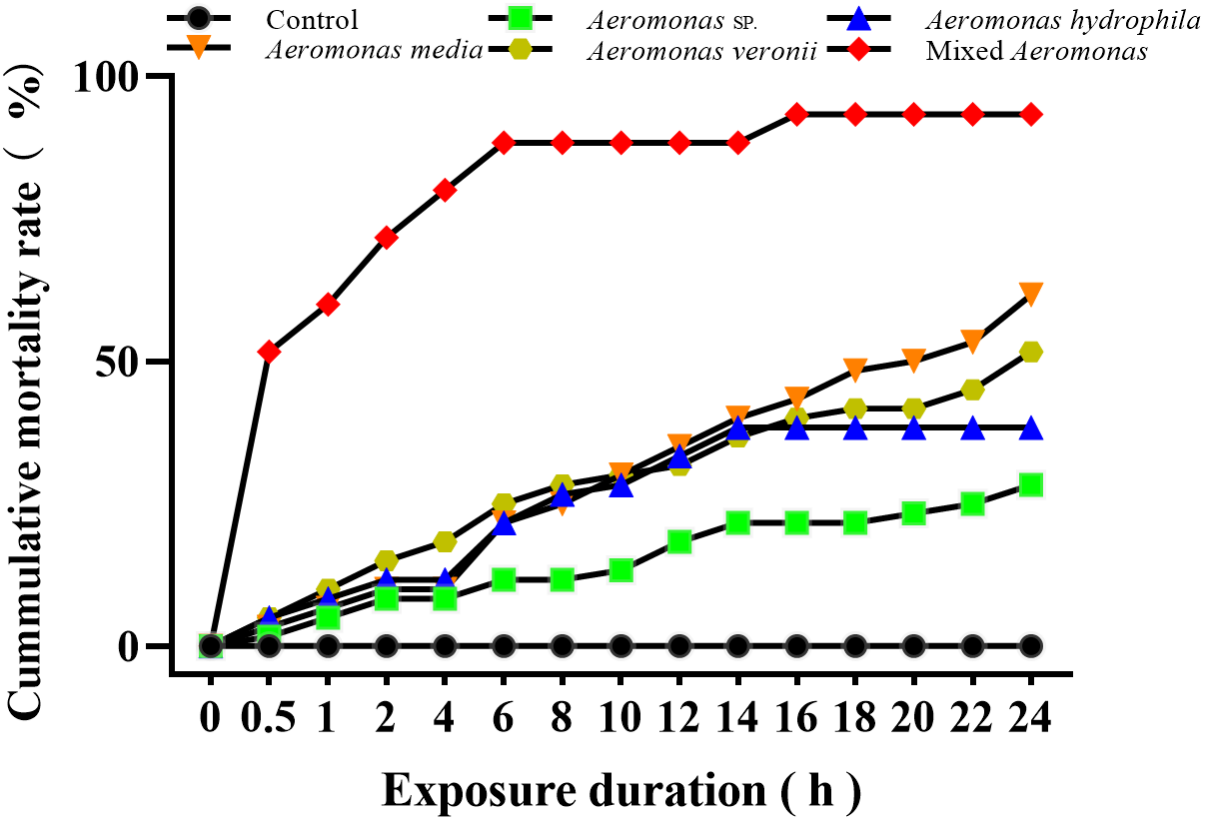
The cumulative mortality rate *L. hoffmeisteri* after exposed to the *Aeromonas* strains isolated from the intestinal tract of *P. canaliculata*.

The 24‐h cumulative mortality rate of *L. hoffmeisteri* with the treatment of *Aeromonas* sp., *Aeromonas hydrophila* , *Aeromonas media* , and *Aeromonas veronii* and mixed *Aeromonas* (As) was 28.3%, 38.3%, 61.7%, 51.7%, and 93.3%, respectively (Figure 6). In contrast, the cumulative mortality rate of *L. hoffmeisteri* in the control group without any *Aeromonas* was 0%. Notably, the mortality rate of *L. hoffmeisteri* exposed with the mixed *Aeromonas* was higher than other groups, which climbed to 60% at 1 h. The interactions among various *Aeromonas* strains originating from the intestinal environment of *P. canaliculata* may result in complex and synergistic effects.

## 4 Discussion

The impact of invasive species on the native ecosystem is multifaceted and profound (Parras and Casadío 2006). The secretions and excreta of *P. canaliculata* contain diverse symbiotic bacteria, among which *Aeromonas* represents a prominent group colonizing the intestinal tract (Chen et al. 2021; Li et al. 2019, 2022). Recent research has demonstrated that the secretions of *P. canaliculata* can release diverse pathogenic bacteria and threat the safety of native aquatic animals (Liu et al. 2024; Sui et al. 2024). *L. hoffmeisteri* is sensitivity to environmental microorganisms, the pathogens released by *P. canaliculata* into their ecosystem are likely a significant factor in the reduction of their populations (Liu et al. 2024). In present study, exposure to non‐free moving *P. canaliculata* has lasting and harmful impacts on *L. hoffmeisteri* . The survival rate of *L. hoffmeisteri* progressively declined within 14 days (Figure 1a), and it was no longer influenced after the removal of *Aeromonas* from the intestinal tract of *P. canaliculata* . Simultaneously, both the mixed strains of *Aeromonas* and *P. canaliculata* significantly reduced the survival rate of *L. hoffmeisteri* (Figure 4a), implying that the release of *Aeromonas* is the primary causative factor in the mortality of *L. hoffmeisteri* induced by *P. canaliculata* .

Exposure to *P. canaliculata* induced alterations in the behavior of migration, swinging, food acquisition, and population identification of *L. hoffmeisteri* . In contrast to the native snail *B. aeruginosa*, exposure to *P. canaliculata* elicited an intensified alarm response to *L. hoffmeisteri* , leading to considerable dispersal from the core population, as well as the diminished motility (Figure 1b–d). Furthermore, exposure to *P. canaliculata* affected the food recognition and population dynamics of *L. hoffmeisteri* , leading to prolonged feeding and integration into larger populations (Figure 1e–h). It has been demonstrated that infection with pathogenic bacteria can alter animal behavior (Swanson et al. 2002), and *Aeromonas* infection can alter fish swimming behavior (Sun et al. 2016). Consistently, AFPC did not affect the behavior of *L. hoffmeisteri* , while *L. hoffmeisteri* from the As and IPC groups displayed similar behavioral patterns to those of the PC group (Figure 4b–h). This finding suggests that *Aeromonas* plays a significant role in shaping the behavioral pattern of *L. hoffmeisteri* . The decline in survival rates and alterations in behavior patterns greatly affects the bioturbation ability of *L. hoffmeisteri* , which may pose the adverse effects on aquatic environment (Nogaro and Burgin 2014; Stief 2013).

Environmental pollution, interspecies interactions, and pathogen infections may induce the generation of reactive oxygen species (ROS) in aquatic animal, inducing to oxidative stress. Invasive species serve as stresses to native populations, leading to extensive oxidative damage in these communities (Leza et al. 2019). Similarly, the current study revealed that the superoxide dismutase (SOD) activity, catalase (CAT) activity, and Malondialdehyde (MDA) content in *L. hoffmeisteri* from the PC group were significantly elevated compared to other groups after 14 days of exposure (Figure 2a,b,d), suggesting that *L. hoffmeisteri* in the PC group has undergone strongly oxidative damage, with the immune system actively engaged in the removal of cellular ROS (Birben et al. 2012; Chen et al. 2017). Excessive ROS can exert cytotoxic effects on cells and lead to damage of proteins, DNA, and lipids, and infection by *Aeromonas* has been associated with the activation of antioxidant enzyme systems and detoxifying processes (Chen et al. 2020). *L. hoffmeisteri* in both the As and IPC groups showed elevated oxidative stress levels, similar to the PC group. However, the AFPC group displayed no signs of oxidative stress, closely resembling the NS group (Figure 5). This suggests that *Aeromonas* infection and exposure to *P. canaliculata* may potentially induce oxidative stress in *L. hoffmeisteri* , thereby influencing their survival and behavior patterns. Furthermore, oxidative stress has a profound impact on the intestinal microbial composition, function and metabolic pathways of the host.

Intestinal microbiota plays a crucial role in the metabolism, immunity, and detoxification of the host, influencing the overall health and normal physiological activities (Dong et al. 2013; Nie et al. 2017; Tremaroli and Bäckhed 2012; Wen et al. 2014). As the primary defense mechanism, the functionality of intestinal microbiota plays a crucial role in the immunity of host (Dong et al. 2013; Nie et al. 2017; Wen et al. 2014). The exposure to *P. canaliculata* resulted in a decrease in the diversity of intestinal microbiota, and leading to infiltration of opportunistic pathogens into the intestine. Exposed to non‐free *P. canaliculata* has been shown to cause a sustained increase in *Aeromonas* of *L. hoffmeisteri* , *Aeromonas* is a common opportunistic pathogen in aquatic environment recognized for eliciting diverse clinical responses in numerous animals (Awan et al. 2018; Parker and Shaw 2011). The PC7d group had a comparatively elevated presence of *Bacteroides* while the abundance in the PC14d group diminished. The bacteria of this genus are closely linked to host intestinal immunity, homeostasis and immune system development (Lanning et al. 2000; Rhee et al. 2004). The PC7d group enhanced the proliferation of *Bacteroides* in *L. hoffmeisteri* , whereas prolonged exposure resulted in the replacement of *Bacteroides* by other microbiota. Exposed to non‐free *P. canaliculata* resulted in a reduction in intestinal microbes associated with nitrogen and phosphate pollution and the treatment of hazardous chemicals, including *P. seudomonas* and *Lactobacillus* (Ramani et al. 2012). This will likely hinder the capacity of bioturbators to eliminate toxins and intensify pollution within the ecosystem. In addition, *Rhodococcus* and Microbacteriaceae, which play a crucial role in host intestinal metabolism by facilitating organic matter degradation, vitamin B synthesis, and pathogen colonization resistance (Hu et al. 2020; Sassera et al. 2013), exhibited extremely low relative abundance in the group exposed to *P. canaliculata* . Consequently, exposure to *P. canaliculata* leads to the colonization of pathogens in the intestine of *L. hoffmeisteri* and a reduction in the abundance of beneficial functional microbiota, potentially altering the overall function of the intestinal microbiota.

## 5 Conclusion

**FIGURE 7:**
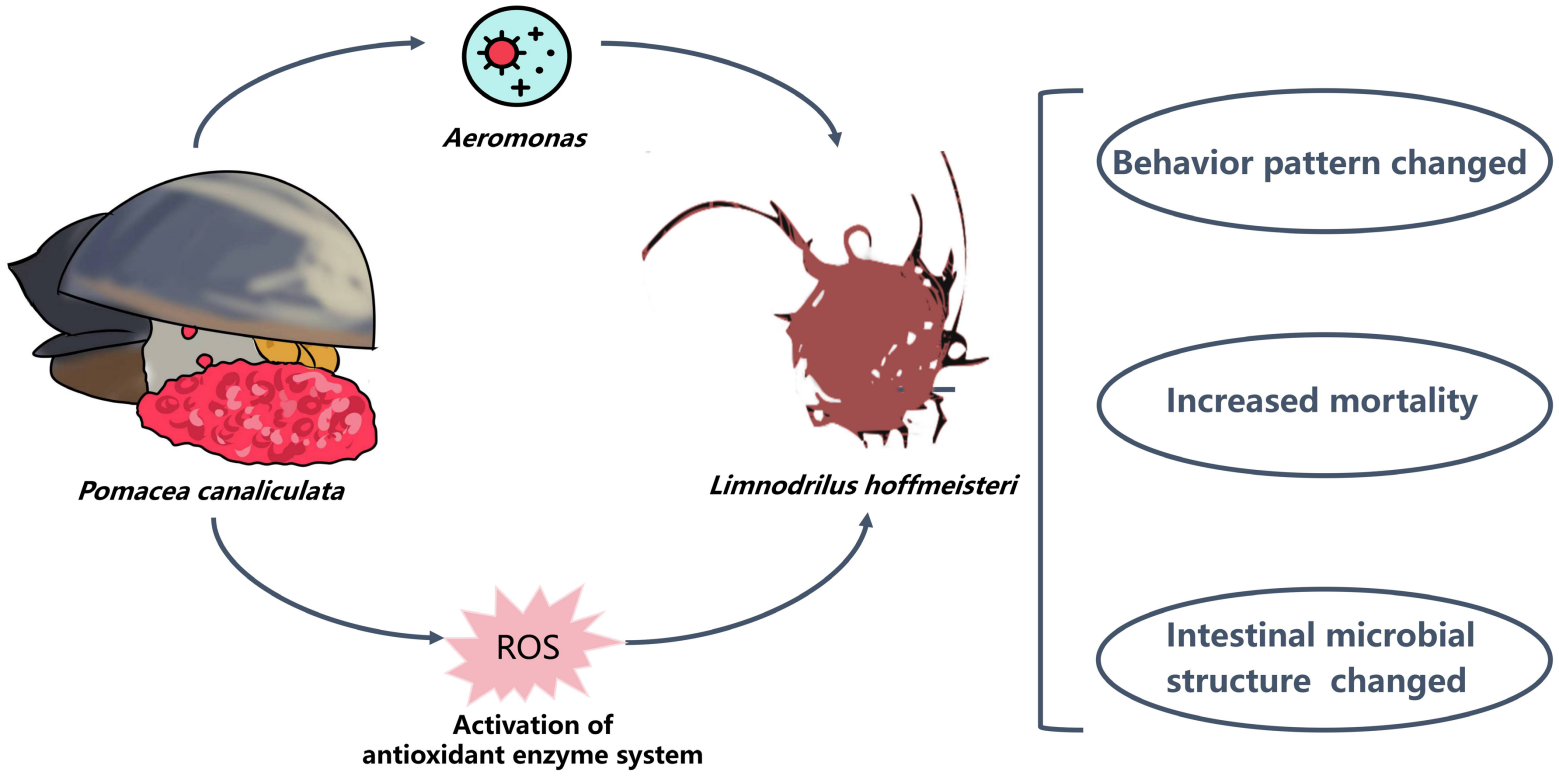
Flow chart of experiment.

Exposure to *Pomacea canaliculata* induces considerable mortality and behavioral alterations in *Limnodrilus hoffmeisteri* . These alterations include a notable reduction in successful food acquisition and aggregation rates, an elevated migration rate, and a diminished swing frequency. These alterations are linked to increased oxidative stress levels in *L. hoffmeisteri* when exposed to *P. canaliculata* . Furthermore, this exposure affects the intestinal microbiota of *L. hoffmeisteri* , leading to a rise in pathogenic colonization, such as *Aeromonas*. *Aeromonas* strains isolated from the intestinal tract of *P. canaliculata* and *Aeromonas*‐reinfected AFPC (IPC) demonstrated analogous patterns in survival, behavior, and oxidative stress in *L. hoffmeisteri* , consistent with the PC group. *P. canaliculata* , of which *Aeromonas* was eliminated from its gut (AFPC), lost the capacity to impact *L. hoffmeisteri* . Consequently, the release of *Aeromonas* by *P. canaliculata* may significantly and extensively harm native benthic organisms (Figure 7). This study elucidates the effects of *P. canaliculata* invasion on native organisms and ecosystems, establishing a theoretical framework for the assessment and restoration of ecosystems invaded by *P. canaliculata* .

## Author Contributions

**Mingyuan Liu:** conceptualization (lead), formal analysis (lead), methodology (lead), project administration (equal), validation (equal), visualization (lead), writing – original draft (lead). **Changrun Sui:** data curation (equal), validation (lead), writing – original draft (equal). **Baolong Wang:** formal analysis (equal), investigation (equal). **Pengfei Ma:** formal analysis (equal), investigation (equal), validation (equal). **Weixiao Zhang:** formal analysis (equal), investigation (equal). **Ruipin Huang:** formal analysis (equal), investigation (equal). **Yuqing Wang:** formal analysis (equal), investigation (equal). **Zhujun Qiu:** formal analysis (equal), investigation (equal). **Wenyu Zhao:** formal analysis (equal), investigation (equal). **Tao Zhang:** formal analysis (equal), investigation (equal). **Qian Zhang:** conceptualization (equal), data curation (equal), funding acquisition (lead), methodology (equal), project administration (lead), resources (lead), supervision (lead), validation (equal), visualization (equal), writing – review and editing (lead). **Ying Liu:** funding acquisition (lead), methodology (equal), project administration (lead), resources (lead), validation (equal), visualization (equal), writing – review and editing (equal).

## Conflicts of Interest

The authors declare no conflicts of interest.

## Data Availability

The datasets presented in this study can be found in the online data repositories: https://doi.org/10.6084/m9.figshare.27283269.
